# Enhanced Antitumor Efficacy and Reduced Systemic Toxicity of Sulfatide-Containing Nanoliposomal Doxorubicin in a Xenograft Model of Colorectal Cancer

**DOI:** 10.1371/journal.pone.0049277

**Published:** 2012-11-07

**Authors:** Jia Lin, Yan Yu, Sarah Shigdar, Ding Zhi Fang, Jun Rong Du, Ming Q. Wei, Andrew Danks, Ke Liu, Wei Duan

**Affiliations:** 1 School of Medicine, Faculty of Health, Deakin University, Waurn Ponds, Victoria, Australia; 2 Department of Biochemistry and Molecular Biology, West China School of Preclinical and Forensic Medicine, Sichuan University, Chengdu, People’s Republic of China; 3 Department of Pharmacology and Biopharmaceutics, West China School of Pharmacy, Sichuan University, Chengdu, People’s Republic of China; 4 School of Medical Science and Griffith Health Institute, Griffith University, Gold Coast Campus, Southport, Australia; 5 Department of Neurosurgery, Monash Medical Centre, Monash University, Clayton, Victoria, Australia; 6 Faculty of Life Sciences, Sichuan University, Chengdu, People’s Republic of China; Stem Cell Research Institute, Belgium

## Abstract

Sulfatide is a glycosphingolipid known to interact with several extracellular matrix proteins, such as tenascin-C which is overexpressed in many types of cancer including that of the colon. In view of the limited success of chemotherapy in colorectal cancer and high toxicity of doxorubicin (DOX), a sulfatide-containing liposome (SCL) encapsulation approach was taken to overcome these barriers. This study assessed the in vitro cytotoxicity, biodistribution, therapeutic efficacy and systemic toxicity in vivo of sulfatide-containing liposomal doxorubicin (SCL-DOX) using human colonic adenocarcinoma HT-29 xenograft as the experimental model. In vitro, SCL-DOX was shown to be delivered into the nuclei and displayed prolonged retention compared with the free DOX. The use of this nanodrug delivery system to deliver DOX for treatment of tumor-bearing mice produced a much improved therapeutic efficacy in terms of tumor growth suppression and extended survival in contrast to the free drug. Furthermore, treatment of tumor-bearing mice with SCL-DOX resulted in a lower DOX uptake in the principal sites of toxicity of the free drug, namely the heart and skin, as well as reduced myelosuppression and diminished cardiotoxicity. Such natural lipid-guided nanodrug delivery systems may represent a new strategy for the development of effective anticancer chemotherapeutics targeting the tumor microenvironment for both primary tumor and micrometastases.

## Introduction

Colorectal cancer is the third most common cause of cancer-related deaths worldwide [1], [2], with up to 25% of patients presenting with metastatic disease. Despite surgery and chemotherapy, many of these patients eventually succumb to metastatic diseases. Adjuvant therapies, including radiotherapy and chemotherapy, are designed to target residual tumor cells. In stage III patients with colorectal cancer, chemotherapy remains the main treatment strategy [3]. However, the success of these therapies is limited by the emergence of therapy-resistant cancer cells as well as dose-limiting toxicities [4]. Over the past few decades, nanoscale therapeutic systems have emerged as novel therapeutic modalities for combating cancer [4]. The nanoparticle formulations of traditional free anticancer drugs may have improved pharmacokinetics and biodistribution profiles, enhanced antitumor efficacy, as well as reduced toxicity to healthy tissues.

Liposomal drugs were the first approved and widely used such nanomedicine for the treatment of cancers [5], [6]. Liposomes are microscopic phospholipid vesicles with a bilayered membrane structure. Preclinical and clinical studies have shown that the pharmacokinetic profiles, as well as the targeting specificities, of liposomes can be controlled and modified to reduce the side effects of encapsulated drugs and enhance their efficacy [7], [8], [9].

We have recently developed a novel liposomal carrier system that is composed of two lipids found in humans, sulfatide and 1,2-dioleoyl-*sn*-glycero-3-phosphoethanolamine (DOPE) [12], [13]. Under physiological pH, DOPE confers stability to sulfatide-containing liposomes (SCL) via its inhibitory effects on liposome fusion, as the incorporation of sulfatide into DOPE vesicles greatly enhances the stability of the liposomes formed, even in the presence of plasma, presumably due to the hydration of the negatively charged sulfate head-group of the glycosphingolipid [10]. We have also demonstrated that the interaction between sulfatide and tenascin mediates the binding of SCL to the ECM and endocytic uptake of the liposomes by tumor cells at least in vitro [10], [11].

The targeted delivery of anticancer agents to the tumor microenvironment is a promising avenue for the therapy of metastatic colorectal cancer. Tenascin-C, a large extracellular matrix hexabracchion glycoprotein, is highly expressed in the microenvironment of most solid tumors, including colorectal cancers, but is absent or greatly reduced in most adult tissues [14]. Our sulfatide-containing liposomal carrier system thus represents a new class of natural lipid-guided intracellular delivery system targeting the tumor microenvironment. In exploring such a new direction for the development of more effective anticancer chemotherapeutics, it is important to understand the in vivo behavior of the nanocarrier, as the unique distribution governed by the properties of the nanocarrier can change the therapeutic efficacy as well as alter the toxicity profile of the encapsulated drug. In this study, we utilize a mouse xenograft model of human colorectal adenocarcinoma (HT-29) that is known to express tenascin-C [15], [16] and a widely used chemotherapy drug, doxorubicin (DOX), as a model payload to study the biodistribution, antitumor efficacy and toxicity of sulfatide-containing liposomal carrier system.

## Materials and Methods

### Ethics Statement

The Deakin University Animal Welfare Committee has approved all animal protocols used in this research.

### Cell Culture

The human colorectal adenocarcinoma cell line HT-29 was purchased from American Type Culture Collection (ATCC, Manassas, VA). McCoy's 5A (modified) medium was purchased from Invitrogen™ (Australia). Fetal bovine serum (FBS) was purchased from Hyclone (Canada). Trypsin was purchased from Invitrogen™ (Australia). Tissue culture flasks were purchased from BD Falcon™ (Australia). Glass bottom dishes were purchased from MatTek Corporation (Ashalnd, MA, US). HT-29 Cells were cultured in McCoy's 5A medium supplemented with 10% fetal bovine serum, penicillin (50 U/mL), and streptomycin (50 µg/mL) in a humidified atmosphere containing 5% CO_2_ and 95% air at 37°C.

### Preparation of SCL-DOX

Liposomes were prepared according to a previously published method with some modifications [10]. Briefly, DOPE unilamellar vesicles containing 30% (molar ratio) sulfatide were prepared by a hydration method followed by polycarbonate membrane extrusion. DOPE (13.35 mM) and sulfatide (6 mM, Avanti Polar Lipids, Inc.) were dissolved in a mixture of chloroform and methanol (2∶1, v/v), and the lipid mixture, composed of DOPE/sulfatide (3∶7, mol/mol), was transferred to glass tubes. Samples were then reduced to a minimum volume under a nitrogen stream, and stored under vacuum for 24 h at 4°C to completely evaporate the organic solvent. The thin lipid films were hydrated by the addition of 1 mL of 250 mM ammonium sulfate (pH 8.5). The samples were then placed in an ice-water bath and sonicated under nitrogen for 2.5 min with 50% amplitude using a sonicator (Sonics & Materials, Inc). Following sonication, the liposomes were formed via extrusion through polycarbonate membranes (Avanti Polar Lipids, Inc.) with consecutive pore sizes of 400 nm for 14 times, 200 nm for 14 times and 100 nm for 19 times at room temperature. To establish a trans-bilayer ammonium sulfate gradient, the extruded liposomes were dialyzed against a 250-fold volume of 10% sucrose in 25 mM Trizma at pH 8.5 at 4°C for 24 h. The external buffer was changed three times during dialysis. After dialysis of the liposomes, DOX, in 10% sucrose at a final concentration of 5 mg/mL, was added to the liposomes at a drug-to-lipid ratio of 0.3∶1 (w/w), followed by incubation in a water bath at 60°C for 1 h. Non-encapsulated DOX was removed by size exclusion chromatography using a Sephadex G-50 column. The concentration of phospholipids (DOPE) in the liposomes was determined as previously described [17]. The vesicle size and zeta potential of SCL were measured using Zetasizer Nano ZS Particle Characterization System from Malvern? Instruments (Malvern, UK). The DOX loaded into SCL was quantified using a fluorescence detector in High Performance Liquid Chromatography (HPLC).

Chromatographic instrumentation was used based on a previously published method with some modifications [18], [19]. Briefly, the HPLC system (Milford, MA, USA) used in this study consists of a Waters e2695 Separation Module and a Waters 2475 Multi λ Fluorescence Detector. The excitation and emission wavelengths were set at the 470 nm and 585 nm, respectively. Chromatographic separation was performed using a Nova-Pak® C18 column (3.9×150 mm i.d., 4 µm, Waters, USA) with a Nova-Pak® C18 guard column (3.9×20 mm i.d., 4 µm, Waters, USA). A mixture of methanol and 10 mM phosphate buffer (pH = 3.0) was used as the mobile phase. The flow-rate used in the assay was 1 mL/min and the column was maintained at 40±5°C throughout the chromatographic process.

### Analysis of Cytotoxicity

The effect of free DOX or SCL-DOX on HT-29 cytotoxicity was determined using the MTT cell proliferation assay [20], [21]. HT-29 cells were seeded at a density of 2×10^3^ cells per well in a 96-well plate in 100 µl McCoy's 5A medium containing 10% FBS. Free DOX solution and SCL-DOX were added to each well 24 h after plating (final concentration 0–100 µg/mL). After 48 h of incubation at 37°C, 5% CO_2_, absorbance was measured at a wavelength of 570 nm using a VICTOR TM X5 Multilabel HTS Plate Reader (PerkinElmer Life and Analytical Sciences). Cytotoxicity was expressed as a percentage of control cells. The inhibition concentration 50% (IC_50_), defined as the dose of agents that inhibited 50% of cell growth, was interpolated from the growth curves using SPSS 13.0 [18], [19]. All experiments were performed in triplicate and repeated thrice.

### Confocal Microscopy Analysis for Cellular Uptake and Retention of SCL-DOX

HT-29 cells (1×10^5^ cells/well) were seeded in 35 mm glass bottom dishes and incubated at 37°C in 5% CO_2_ for 24 h. The medium was then replaced with full culture medium containing 2 µg/mL free DOX or SCL-DOX. Twenty-four hours later, cells were washed twice with phosphate buffered saline (PBS) and imaged for cellular uptake studies. For retentions studies, cells were first exposed to 2 µg/mL free DOX or SCL-DOX in full cell culture medium for 24 h and then washed twice with PBS. Cells were then incubated with fresh cell culture medium and serially imaged at 1 h, 2 h, 4 h, and 24 h using a Fluoview FV10i fluorescence laser scanning confocal microscopy (Olympus, Japan).

### Analysis of Pharmacokinetic Properties in vivo

Male Sprague-Dawley (SD) rats (200 to 250 g) were housed in a temperature controlled room (25±1°C) with a 12-h light-dark cycle. Rats were fed *ad libitum* with a standard diet but were fasted overnight before free DOX or SCL-DOX administration. All procedures involving animal experimentation were approved by the Deakin University Animal Welfare Committee.

To investigate the pharmacokinetics (PK) properties of SCL-DOX in vivo, healthy SD rats were injected intravenously with free DOX or SCL-DOX via the tail vein with a single dose of 5 mg DOX/kg. Blood was serially collected from the same animal in heparinised tubes from the tail at 2 min, 0.5 h, 2 h, 6 h, 24 h and 48 h. After collection, samples were centrifuged at 3,000×*g* at 4°C for 10 min to separate the plasma. To determine DOX levels in plasma, 495 µl of methanol and 405 µl of phosphate buffer were then added to 100 µL plasma, vortexed for 1 min, and centrifuged at 21,000×*g* for 10 min at 4°C. The supernatant was transferred to another tube followed by the addition of 2 µL of perchloric acid (35%, v/v). The samples were vortexed for 1 min, and centrifuged at 21,000×*g* for 10 min at 4°C, followed by the measurement of DOX concentration using HPLC.

### Tumor Implantation, Treatment and Evaluation

Xenograft tumors were established in 6 weeks old female BALB/c-Foxn1^nu^ mice that were purchased from The Animal Resources Centre (Perth, Australia). All animal experiments were performed in accordance with the guidelines of institutional Animal Welfare Committee of Deakin University. Mice were kept under pathogen-free conditions in TECNIPLAST Sealsafe™ Individually Ventilated Cages (Buguggiate, Italy) at (25±1°C) and a 12-h light/12-h dark cycle. They were fed *ad libitum* with a standard diet.

HT-29 cells used for xenograft tumors were prepared by trypsinization. The cells were washed and resuspended at a concentration of 3×10^7^ cells/mL in PBS, which was then inoculated subcutaneously (s. c.) into the right flank of the mice. Tumor size was assessed using a digital caliper every other day after implantation and approximate tumor burden (mm^3^) was calculated as length×width^2^/2 (*V* = *lw* ^2^/2), where length and width are the longest and shortest axis in millimeters [22].

For the tumor uptake study, mice with tumors of ∼150 mm^3^ were treated with free DOX or SCL-DOX (5 mg/kg DOX or equivalent) via tail vein injection. Twenty-four hours after injection, mice were sacrificed by injection of Lethabarb R (100 mg/kg) and tumors were processed as previously described [23], [24]. DOX concentration in tissue was determined using HPLC.

For therapeutic experiments, mice were treated when the xenograft tumors reached 35 mm^3^. Mice received an injection of saline, free DOX (5 mg/kg), SCL-DOX (5 mg/kg in DOX) or blank SCL via tail vein twice a week for 3 weeks. Tumor growth was monitored by measuring tumor diameters every other day with a caliper and animal weights were monitored at the same time. The end point of this study was defined as the tumor load reaching 1700 mm^3^.

### Analysis of Systemic Toxicity

To evaluate the general toxicity of free DOX and SCL-DOX, blood was collected when the mice for therapeutic experiments were sacrificed. Blood cells counts and troponin were analyzed by a veterinary pathology laboratory (Gribbles Veterinary Pathology, Clayton, Victoria, Australia). Blood smears were obtained for each animal to obtain a relative white cell count adapted from the Fonio method for platelet counting [25], [26], with minor modifications. Slides were stained with Giesma and an area of the blood smear was chosen where the red cells abutted each other but did not overlap, with consecutive fields chosen to eliminate bias. The total number of white cells per 1500 red cells were counted (n = 3 for each slide) and compared for each group.

### Data Analysis

All the results are presented as means and standard error (mean±S.E.). The pharmacokinetic parameters were calculated from the average plasma concentrations using the pharmacokinetic software DAS 2.0 software (Mathematical Pharmacology Professional Committee of China, Shanghai, China). The differences in the mean values among different groups were determined by a one-way analysis of variance (ANOVA) using SPSS 13.0 program. Significance was considered at values of *p*<0.05.

## Results

### Characterization of SCL

The diameter of SCL incorporating DOX was found to range within 92.3±1.3 nm (mean±S.E.; n = 10) with polydispersity index (PDI) of 0.15±0.01 (mean±S.E.). At an initial weight ratio of DOX to DOPE of 0.3∶1, the SCL had an average DOX entrapment efficiency of 94.11±2.27% (mean±S.E). The zeta potential value of SCL was −26.38±2.20 mV (mean±S.E.). The DOX to DOPE weight ratio after DOX encapsulation into SCL was 0.5∶1.

### Intracellular Uptake and Retention of SCL-DOX in HT-29 Cells

**Figure 1:**
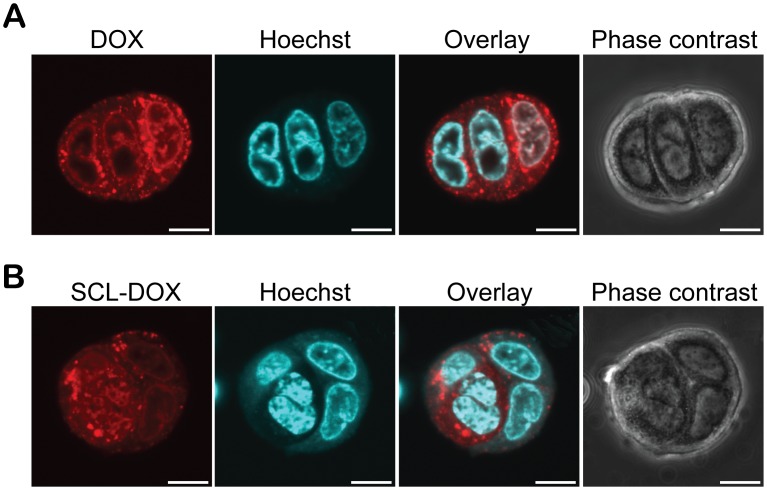
Intracellular uptake of SCL-DOX in HT-29 cells. HT-29 cells were incubated with 2 µg/mL free DOX or equivalent SCL-DOX for 24 h. Following two washes with PBS, cells were imaged with a confocal fluorescence microscope. (**A**) Cells treated with free DOX. (**B**) Cells treated with SLC-DOX. Red: fluorescence from DOX; blue: nuclei stained with Hoechst 33342. Scale bars: 10 µm.

**Figure 2:**
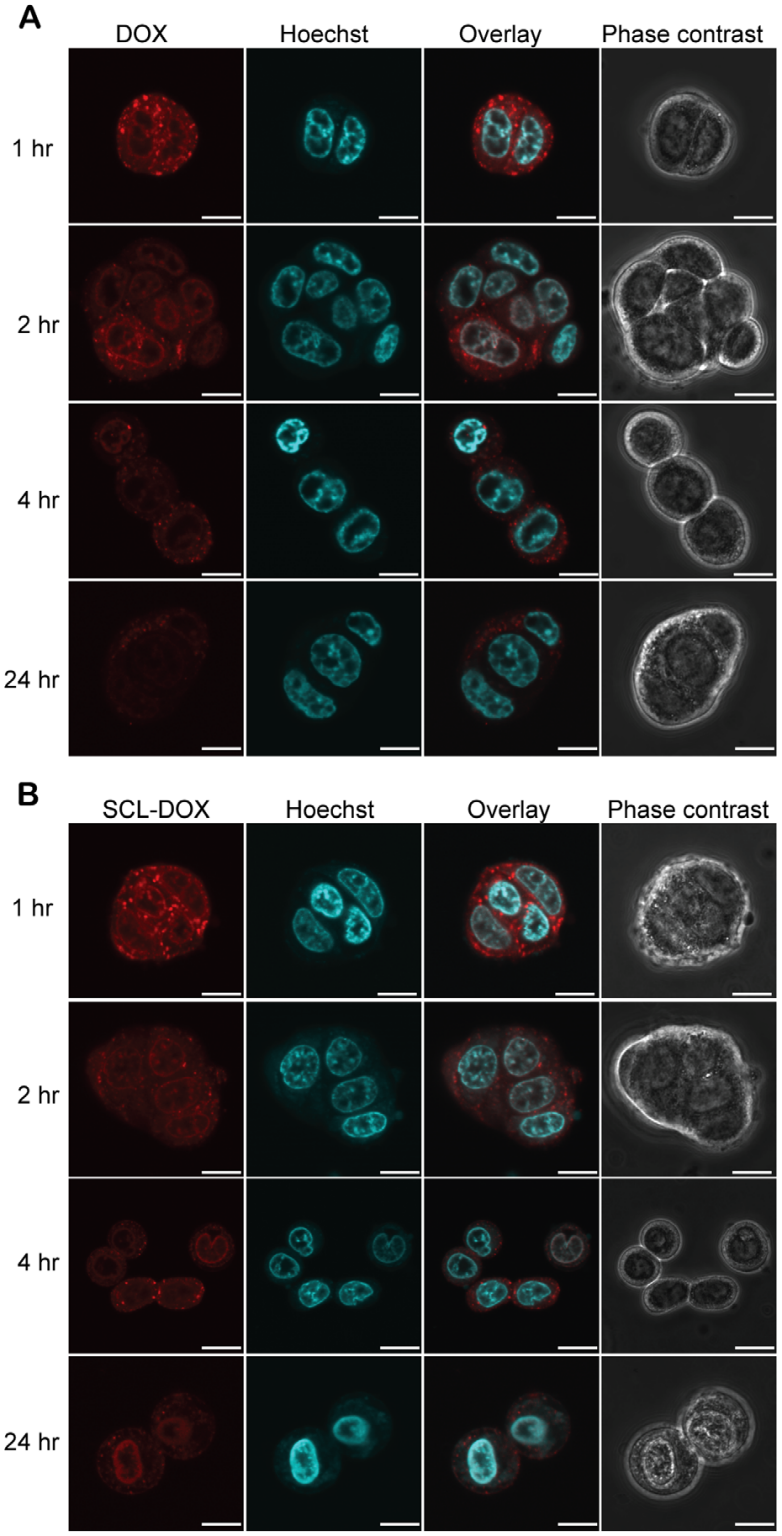
Intracellular retention of SCL-DOX in HT-29 cells. HT-29 cells were first incubated with 2 µg/mL free DOX or equivalent SCL-DOX for 24 h. After two washes with PBS to remove the drugs, cells were cultured in fresh full culture medium followed by imaging serially at 1 h, 2 h, 4 h and 24 h using fluorescence confocal microscopy. (**A**) Cells treated with free DOX. (**B**) Cells treated with SLC-DOX. Red: fluorescence from DOX; blue: nuclei stained with Hoechst 33342. Scale bars: 10 µm.

Taking advantage of the natural fluorescent property of DOX, the cellular uptake and retention of free DOX or SCL-DOX was studied using laser scanning confocal microscopy. HT-29 cells were incubated with 2 µg/ml DOX or SCL-DOX for 24 h. Following washing, the cellular uptake of different formulations of DOX was examined. As shown in Figure S1 (low magnification) and Figure 1 (high magnification), both free DOX and SCL-DOX were taken up by the colorectal adenocarcinoma cells and there were accumulations of DOX in the nucleus in both groups (Figure 1), albeit cells treated with free DOX showed slightly stronger red fluorescence (DOX) than those treated with SCL-DOX after 24 h incubation. Interestingly, the retention of SCL-DOX in HT-29 cells was better than that for free DOX. As shown in Figure S2A (low magnification) and Figure 2A (high magnification), following washing with PBS and incubation in fresh media for 4 h, the DOX fluorescence in the free DOX group decreased significantly. Moreover, cells treated with free DOX showed diminished red fluorescence 24 h after washing. Conversely, the red fluorescence for SCL-DOX was more stable compared to that of the free DOX group. Even 24 h after washing, DOX fluorescence could be readily observed in the nuclei of cells treated with SCL-DOX (Figure S2B and Figure 2B). The enhanced retention of SCL-DOX in vitro suggests that the SCL formulation of DOX might exhibit better treatment efficacy in vivo.

### In vitro Cytotoxicity

To study the in vitro cytotoxicity, HT-29 cells were exposed to various concentrations of free DOX or SCL-DOX for 48 h, and the cell viability was measured using the MTT assay. As shown in Table 1, the IC_50_ of DOX for HT-29 cells was 1.74±0.10 µg/mL while the IC_50_ of SCL-DOX was 2.77±0.06. Thus, under in vitro conditions where cells were exposed to a constant concentration of the agents throughout the entire assay period, free DOX was more toxic than SCL-DOX. Empty SCL did not show any effects on cell survival (data not shown).

**Table 1:** Mean IC_50_ values (µg/ml of doxorubicin) for treatment with free DOX and SCL-DOX.

Cell line	Free dox (µg/ml)	SCL-dox (µg/ml)
HT-29	1.74±0.10	2.77±0.06

Data are shown as means±S.E. of triplicate in three independent experiments.

### Improved Pharmacokinetic Properties of SCL in Healthy SD Rats

The pharmacokinetic properties of both free DOX and SCL-DOX were studied in healthy male SD rats. The serum clearance kinetics of free DOX and SCL-DOX was compared as shown in Table 2. In our study, the clearance rate of DOX encapsulated by SCL-DOX (1.39 L/h/kg) was significantly lower than that of DOX solution (2.68 L/h/kg, *p*<0.01), suggesting a different rate of clearance of SCL-DOX compared to free drug. Moreover, the area under the plasma concentration-time curves during the study period (AUC_0–48 h_) of DOX delivered through SCL was 2.37-fold higher than free DOX (*p*<0.01). Thus, DOX could display a significantly reduced clearance rate as well as enhanced bioavailability when administered entrapped in SCL.

**Table 2:** Comparison of pharmacokinetic parameters of free DOX and SCL-DOX in SD Rats.

	Formulations	
Parameter	Free DOX	SCL-DOX
Dox dose (mg/kg)	5	5
AUC_0–48_ (µg/Lh)	1358.00±28.55	3264.62±79.65**
CL (L/h/kg)	2.68±0.22	1.39±0.04**

Pharmacokinetic parameters were calculated after the i.v. injection of free DOX or SCL-DOX in healthy SD rats at a dose of 5 mg/kg. Data are shown as means±S.E. of at least three independent experiments.

AUC_0–48_: Area under the plasma concentration-time curve.

CL: Total body clearance.

** , *P*<0.01 compared to free DOX.

### Biodistribution and Tumor Uptake Advantages of SCL-DOX

**Figure 3:**
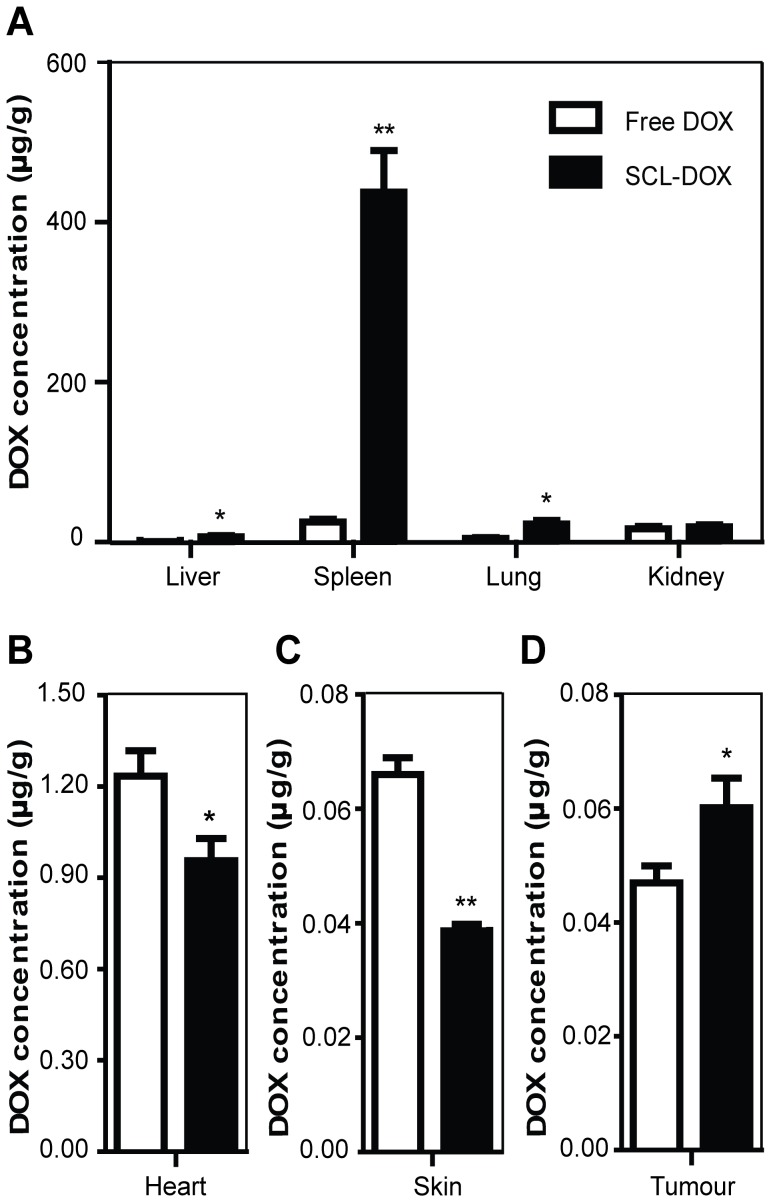
Biodistribution and tumor uptake of doxorubicin encapsulated in sulfatide-containing liposome in xenograft-bearing mice. Nude mice bearing human colorectal cancer HT-29 xenografts were treated with 5 mg/kg free DOX or SCL-DOX i.v. Mice were euthanized 24 h later. Organs and tissues were harvested, washed, weighed, and the DOX was extracted and quantified. Data are shown as means ± S.E. (n = 5∼6). *, *P*<0.05 compared to free DOX; **, *P*<0.01 compared to free DOX.

Studies comparing the accumulation of free DOX or SCL-DOX in tumors and organs were performed in a BALB/c nude mice HT-29 tumor xenograft model. Animals were injected i.v. with a single dose of free DOX or SCL-DOX (5 mg/kg) and there was no statistically significant difference in DOX concentration in the kidneys between the two treatment groups 24 h after administration. The lungs and liver showed higher DOX accumulation (4.74-fold and 12.94-fold, respectively) with SCL-DOX treatment (Figure 3A), and the spleen, a major organ of the reticuloendothelial system, showed a 17-fold higher DOX accumulation with SCL-DOX treatment. However, SCL-DOX treatment in the two principal organs that display dose-limiting toxicities of DOX clinically, namely the skin and heart, decreased the DOX accumulation to 59.0% (0.039±0.001 µg/g versus 0.066±0.003 µg/g) and 77.4% (0.956±0.073 µg/g versus 1.235±0.083 µg/g) compared to the free DOX, respectively (Figure 3A and 2B). Moreover, SCL encapsulation significantly enhanced DOX accumulation (1.3-fold; 0.060±0.005 µg/g versus 0.047±0.003 µg/g) in the xenograft tumor compared to the free DOX (Figure 3D), clearly confirming the enhanced intratumoral DOX delivery by SCL-DOX in vivo.

### Enhanced Therapeutic Efficacy of SCL-DOX

**Figure 4:**
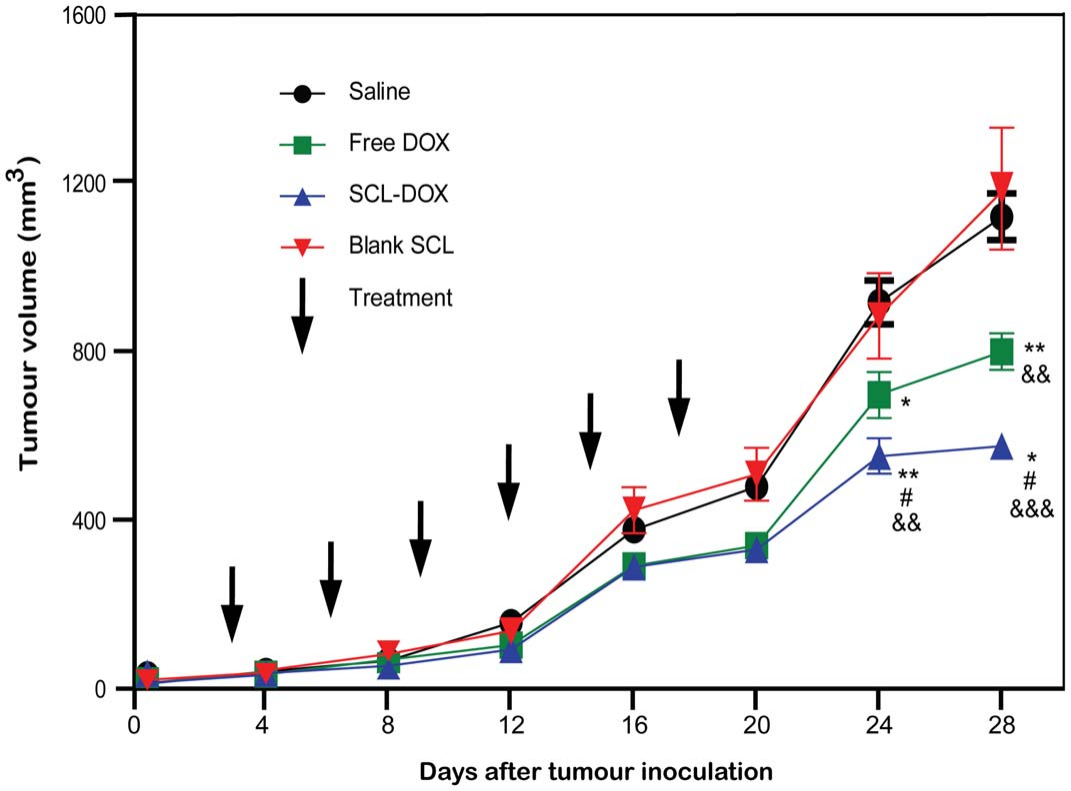
Sulfatide-containing liposomal doxorubicin produced more reduction in tumor volume. Mice bearing HT-29 xenografts were injected i.v. with saline, 5 mg/kg of free DOX, SCL-DOX or empty SCL twice a week for 3 weeks as indicated, starting on the day when tumor volume reached ∼35 mm^3^. Data shown are means ± S.E. (n = 5∼6). *, *P*<0.05 compared to saline; **, *P*<0.01 compared to saline; #, *P*<0.05 compared to free DOX; &&, *P*<0.01 compared to blank SCL; &&&, *P*<0.001 compared to blank SCL.

We evaluated the antitumor activity of SCL-DOX using the BALB/c nude mice HT-29 tumor xenograft model. Once the tumor had grown to approximately 35 mm^3^, we divided the animals randomly into four groups (n = 5∼10) in order to minimize difference in weight and tumor size among the groups. The following regimens were administered i.v. twice a week for 3 weeks: (*i*) saline; (*ii*) empty SCL; (*iii*) free DOX (5 mg/kg) and (*iv*) SCL-DOX (5 mg/kg). The body weight of the animals and the tumor size were then monitored until the size of the tumor in the control animals reached the end point of the study. As presented in Figure 4, for the control groups of mice receiving saline or empty SCL, the treatment did not show any efficacy, and the mean tumor sizes at the end of the study were 1129.03±55.06 mm^3^, and 1188.63±137.54 mm^3^, respectively (mean ± S.E.; n = 5∼6). The SCL-DOX treatment group demonstrated superior efficacy, with a final mean tumor load of 586.52±29.63 mm^3^, compared to 809.13±43.75 mm^3^ in the free DOX group. Thus, compared to saline or free DOX treatment, the efficacy of SCL-DOX to suppress tumor growth at the dose of 5 mg/kg was significantly improved by ∼1.9-fold and ∼1.4-fold, respectively.

**Figure 5:**
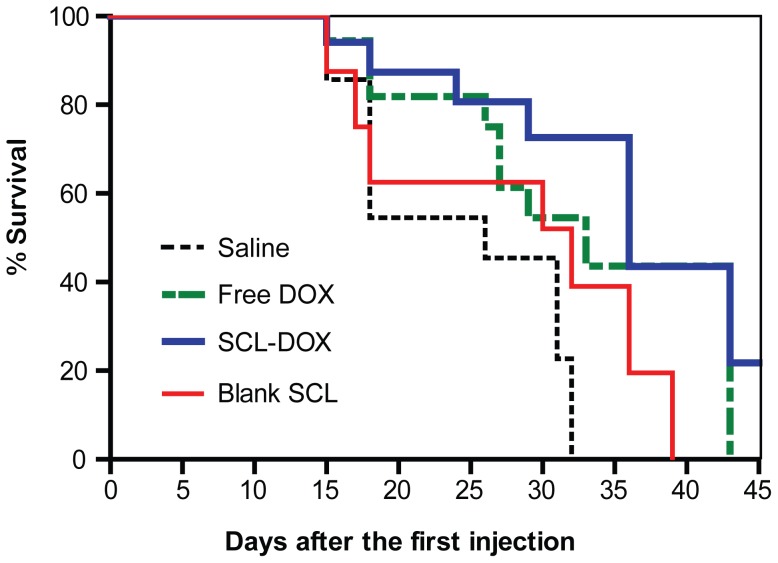
Sulfatide-containing liposomal doxorubicin enhanced survival. The Kaplan-Meier survival curve shows improvement of life span of xenograft-bearing mice treated with SCL-DOX (n = 9∼10 per group). Mice were treated as indicated in Figure 3 and were sacrificed throughout the study period upon reaching our study end point.

Next, we compared the survival rates of tumor-bearing mice following the four different treatment regimens. As shown in Figure 5, median survival times for the four different groups were 26 days (saline), 33 days (free DOX), 36 days (SCL-DOX) and 32 days (blank SCL), respectively. Thus, SCL-DOX treatment increased medium life-span by 38.5% compared to the saline control group, by 12.5% compared to the blank SCL group and by 9.1% compared to the free DOX group. These experiments demonstrated that the administration of SCL-DOX in 6 doses over a three-week period not only afforded better inhibition of tumor growth but also improved the survival of xenograft-bearing animals.

### Reduced Systemic Toxicity of SCL-DOX

**Figure 6:**
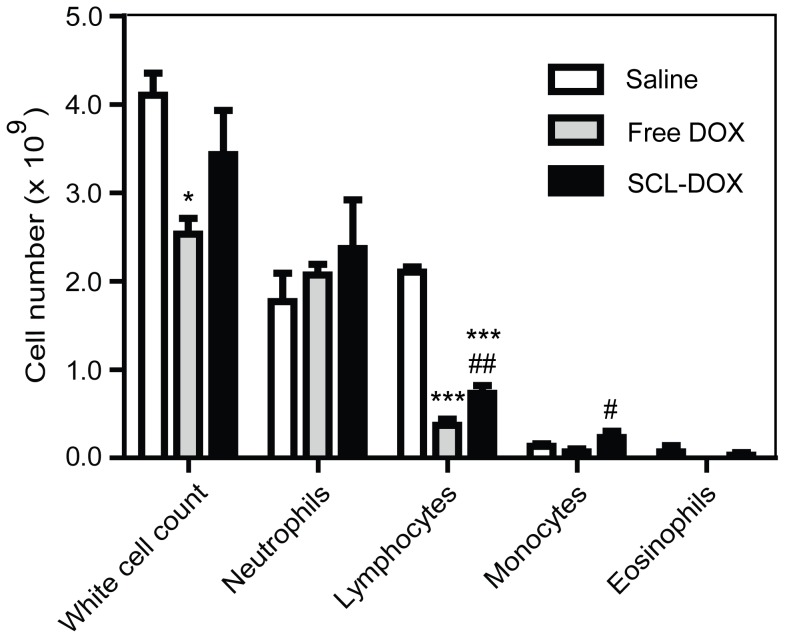
Sulfatide-containing liposomal doxorubicin treatment had significantly reduced myelosuppression. HT-29 xenograft-bearing mice were treated as indicated in Figure 3. Blood was collected immediately after the mice were sacrificed upon reaching the end point. Data shown are means ± S.E. (n = 3∼5). *, *P*<0.05 compared to saline; ***, *P*<0.001 compared to saline; #, *P*<0.05 compared to free DOX; ##, *P*<0.01 compared to free DOX.

DOX-induced cardiomyopathy is one of the key dose-limiting toxicities of the drug [27]. Cardiac troponin-T is released from DOX-damaged myocytes [28], therefore, measurement of serum levels of this protein provides a sensitive assessment of early cardiotoxicity of DOX. The method used in the present study has a cut-off threshold of <0.01 µg/L for normal subjects [29]. As shown in Table-3, the free DOX treatment resulted in a 75-fold higher troponin serum level compared to the controls, confirming the known cardiotoxicity of the free drug. However, no elevation of serum troponin was observed for the SCL-DOX treatment group, which remained below cut-off levels, as with the control groups, indicating that treating xenograft-bearing mice with 6 doses of SCL-DOX over the period of 4 weeks had minimal cardiotoxicity. To investigate whether encapsulation of DOX into SCL had any impact on the severity of bone marrow suppression (myelosuppression), the most common adverse effect of DOX chemotherapy [27], we studied the changes in peripheral white blood cells count. As shown in Figure 6, there was no statistically significant difference in the total number of white blood cells between the saline treated or SCL-DOX-treated groups. Furthermore, compared with mice treated with free DOX, those treated with SCL-DOX had a 2.0-fold and a 3.3-fold higher count for lymphocytes and monocytes, respectively. Thus, our data suggest that SCL-DOX has minimal cardiotoxicity and significantly reduced myelosuppression.

**Table 3:** Plasma levels of troponin.

Treatment	Number of nude mice	Troponin (ug/L)
Saline	4	<0.01
Free DOX	3	0.750±0.234
SCL-DOX Blank SCL	5 3	<0.01<0.01

Data are presented as means±S.E. (n = 3–5).

## Discussion

This study is the first to evaluate the tissue distribution, in vivo anticancer activities and toxicity profile of SCL-DOX in a xenograft mouse model of human colorectal adenocarcinoma. We have shown several important points: (a) SCL-DOX was readily taken up by colon cancer cells and displayed prolonged retention; (b) encapsulation of DOX in SCL resulted in a decreased distribution of the drug to the principal sites of acute and chronic toxicity of free DOX, namely the heart and the skin, as well as markedly lowered cardiotoxicity and myelosuppression; (c) sulfatide-containing liposomal drug displayed an enhanced therapeutic efficacy in a mouse model of human colorectal adenocarcinoma.

The intracellular uptake of SCL in glioma cells was shown to be a result of endocytic uptake of the liposomes in previous work [11]. In this study, we confirmed the intracellular uptake of SCL-DOX by human colorectal adenocarcinoma cells, HT-29, using confocal microscopy. Importantly, we demonstrated that the encapsulated chemotherapy drug was delivered intracellularly to the site of action, the nuclei, of the HT-29 cells (Figures 1 and 2). Furthermore, the delivered liposomal drug was retained by the tumor cells even 24 h after washing. Our in vitro cytotoxicity study compared the viability of colorectal cancer cells treated with SCL-DOX and free DOX using the MTT assay and showed that both forms possess overt cytotoxicity. Interestingly, the SCL-DOX has an IC_50_ 59% higher than that of free DOX (Table 1). This is consistent with observations from others that the IC_50_ of the free and liposomal drug, when assayed in vitro, varies dependent on the cell lines used and the nature of the liposomes. For example, Wang et al. found in the rat prostate cancer cell line MLLB2, the IC_50_ of the liposomal formulation was significantly lower than free DOX [30]. In a study of resistant MCF-7/ADR cells, the liposomal DOX showed a 30-fold lower IC_50_ compared to free DOX [31]. However, in other studies free DOX seems to have higher intracellular uptake and displays higher cytotoxicity than that of liposomal DOX. For example, in the hepatocellular carcinoma cell line, HepG2, free DOX has been indicated to possess a higher cytotoxicity compared to that of DOX-loaded stealth liposomes [24]. Furthermore, polyethylene glycol (PEG) coated-liposomal DOX has been shown to have less toxicity than free DOX in the glioma cell line, U-87 cells [10]. It is important to appreciate that the in vivo pharmacokinetics are very different between liposomal DOX and free DOX. The half-life of liposomal DOX can be several days while free DOX can be eliminated in a few minutes in vivo [32], [33], [34]. In cell culture dishes, cells are exposed to a constant drug concentration throughout the entire assay period, and often take up free DOX more rapidly than liposomal formulation [35]. Furthermore, cells for MTT assays are mostly cultured in monolayers, which have a spatial organization drastically different from the in vivo 3-dimensional tissue architecture [36]. Thus, comparison of IC_50_ between a free drug and a nanoparticle-formulation of the drug in vitro provides a measurement of the cytotoxicity under a constant concentration during a chosen assay period, and therefore it may not be able to provide a reliable prediction of the therapeutic efficacy in vivo [37]. In the present study, although the IC_50_ of SCL-DOX was higher than that of free DOX in HT-29 cells in vitro, the SCL formulation was shown to display a much improved tumor inhibitory effect over the free DOX in HT-29 tumor-bearing nude mice (Figures 4 and 5).

Gastrointestinal tumors are known to be relatively resistant to chemotherapeutics. In 80% of untreated colon tumors, there is an elevated level of multidrug-resistant I (MDR I) gene [38]. The comparison of pharmacokinetic properties between the free DOX and SCL-DOX in healthy SD rats displayed the significant decreased of clearance rate of SCL-DOX compared to free DOX (*p*<0.01). The same improvement was found in the area under the plasma concentration-time curve (Table 2), indicating reduced clearance rate and enhanced bioavailability of our SCL-DOX. Notably, significantly higher concentrations of DOX in tumor from SCL-DOX treated mice were found after single administration (*p*<0.05). Moreover, it is interesting to note that SCL encapsulation not only resulted in a significant increase in tumor uptake in vivo, but also enhanced the anti-tumor efficacy of DOX in HT-29 tumor-bearing nude after repeated administration. As the statistically significant difference in tumor growth between free DOX and SCL-DOX groups appeared after the 5th administration (Figure 4), the therapeutic efficacy may be attributed to the preferential accumulation of SCL-DOX in the tumor via the enhanced permeability and retention effect over the period of multiple administrations. Moreover, after intracellular delivery, the SCL-DOX was retained in the tumor cells much better than free DOX (Figure 2). Thus, the increased DOX concentration in tumors treated with SCL-DOX is consistent with a better treatment efficacy in vivo.

In most cases, cancer chemotherapy is limited by the low therapeutic index of the anticancer drugs due to serious toxicity to normal tissues. Indeed, the therapy-limiting toxicity of DOX is cardiomyopathy, which may lead to congestive heart failure and death [27]. Encapsulation of free DOX using a cyanoacrylate nanoparticle shifted toxicity from the heart to the kidney [39], while the PEGylated liposomal DOX (Doxil) accumulates in skin, resulting in a shift in the toxicity profile from cardiotoxicity and myelosuppression to cutaneous toxicity, known as palmar-plantar erythrodysesthesia [40]. We aimed to develop a nanoliposome that improves the biodistribution characteristics and changes the toxicologic properties of the encapsulated drug. In our preclinical toxicology experiments, we utilized the intended clinical administration route, i.e. intravenous injection, throughout the studies with the HT-29 tumor-bearing mice models. Our data (Figure 3) demonstrated that the accumulation of liposomal DOX in the skin and heart were significantly lower than that of free DOX. Since tissue levels of DOX were reduced by SCL delivery, the decreased concentration of DOX at these sites is likely to result in a reduced risk of the development of side effects. Indeed, the biochemical and haematological analyses (Figure 6 and Table 3) demonstrated that the improved therapeutic efficacy of SCL-DOX was obtained without an increase in toxicity to the heart or to the bone marrow. It is noteworthy that the troponin level was significantly lower in the SCL-DOX group than that of the free DOX group. As for the indicators of myelosuppression, the higher level of total white cell numbers, lymphocytes and monocytes in the SCL-DOX group indicated that formulation via SCL decreased the toxicity of myelosuppression of the encapsulated drug. The increased accumulation of SCL-DOX in the liver, spleen and lung compared to the free DOX may be related to the particle size of SCL, as nanoparticles with sizes of 100–200 nm preferentially accumulate in organs of the reticuloendothelial system [41], [42], [43].

By encapsulating drugs in nanoparticles, nanomedicine reduces the drug concentration in normal host tissues and increases the concentration of active drug within the tumor. To further improve the selectivity and specificity, it is desirable to actively target the nanodrugs to the site of the tumor. Targeting nanodrugs using antibodies that recognize the tumor-associated antigens is a widely adopted approach. However, its application might be limited by altered expression or low percentages of tumor cells that express any given antigen. Therefore, the development of alternative targeted drug delivery systems may have important implications for future novel anticancer therapeutics. We have previously demonstrated that sulfatide mediates the binding and endocytic uptake of SCL in tumor cells via the interaction with tenascin-C [11]. The extracellular matrix is a major constituent of the tumor microenvironment that is very different from that of their normal counterparts. Tenascin-C is a large extracellular matrix hexabrachion glycoprotein and is absent or greatly reduced in most normal adult tissues. Tenascin-C is highly expressed in the majority of malignant solid tumors, including gliomas, and cancer of the breast, uterus, ovaries, prostate, colon, stomach, pancreas, lung, liver, skin and kidney [14]. Furthermore, high tenascin-C expression correlates with a low survival prognosis in cancers such as glioma, breast, colon and lung carcinoma. In addition to its roles in promoting tumor cell proliferation and angiogenesis, tenascin-C has recently been shown to provide breast cancer cells with a key metastatic niche to colonize the lungs by promoting tumor cell dissemination and survival during the early steps of metastasis and enhancing the fitness of the disseminated cancer cells at the site of colonization [44]. Thus, the production of tenascin-C enhances the ability of micrometastatic colonies to survive and expand. Given that up to 20% of new cases of colorectal cancer present with metastatic disease, and of the patients who present with localized disease, about 20% will subsequently relapse with distant metastases [4], targeting tenascin-C would constitute a promising strategy in our effort to combat micrometastasis. Sulfatide has been shown to bind to several extracellular matrix proteins, including tenascin-C [45], [46]. In this study, we further demonstrated the in vivo utilities of the SCL in enhanced efficacy in colorectal cancer xenografts known to express tenascin-C as well as reduced concentration and toxicity in tissues that are susceptible to the main side effects of the encapsulated drug, i.e. the heart, the bone marrow and the skin. Future research in combining active targeting ligands, such as antibodies and aptamers, which target cancer stem cell surface makers with natural lipid-guided intracellular delivery, may open a new direction for the development of more effective anticancer nanotherapeutics.

In conclusion, we have demonstrated that SCL-DOX showed an improved toxicity profile and enhanced efficacy in a human colorectal cancer xenograft model. It may therefore provide a potent and safe nanomedicine platform for treatment of cancer that has overexpression of sulfatide-binding proteins, especially tenascin-C. Functionalizing SCL with antibodies or aptamers may further enhance the clinical utilities of this natural lipid-guided liposomal formulation of anticancer drugs for both primary tumor and micrometastasis via effective targeting the tumor microenvironment.

## Supporting Information

Figure S1 **Intracellular uptake of SCL-DOX in HT-29 cells.** HT-29 cells were incubated with 2 µg/mL free DOX or equivalent SCL-DOX for 24 h. Following two washes with PBS, cells were imaged with a confocal fluorescence microscope. (**A**) Cells treated with free DOX. (**B**) Cells treated with SLC-DOX. Red: fluorescence from DOX; blue: nuclei stained with Hoechst 33342. Scale bars: 200 µm.
(TIF)

Figure S2 **Intracellular retention of SCL-DOX in HT-29 cells.** HT-29 cells were first incubated with 2 µg/mL free DOX or equivalent SCL-DOX for 24 h. After two washes with PBS to remove the drugs, cells were cultured in fresh full culture medium followed by imaging serially at 1 h, 2 h, 4 h and 24 h using fluorescence confocal microscopy. (**A**) Cells treated with free DOX. (**B**) Cells treated with SLC-DOX. Red: fluorescence from DOX; blue: nuclei stained with Hoechst 33342. Scale bars: 200 µm.
(TIF)

## References

[1] Sala-Vila A, Calder PC (2011) Update on the relationship of fish intake with prostate, breast, and colorectal cancers. Crit Rev Food Sci Nutr 51: 855–871. 21888535 10.1080/10408398.2010.483527

[2] Szepeshazi K, Schally AV, Halmos G, Armatis P, Hebert F, et al (2002) Targeted cytotoxic somatostatin analogue AN-238 inhibits somatostatin receptor-positive experimental colon cancers independently of their p53 status. Cancer Res 62: 781–788. 11830533

[3] Bhattacharya A, Tóth K, Sen A, Seshadri M, Cao S, et al (2009) Inhibition of colon cancer growth by methylselenocysteine-induced angiogenic chemomodulation is influenced by histologic characteristics of the tumor. Clin Colorectal Cancer 8: 155–162. 19632930 10.3816/CCC.2009.n.025 PMC2823082

[4] Segal NH, Saltz LB (2009) Evolving treatment of advanced colon cancer. Annu Rev Med 60: 207–219. 19630571 10.1146/annurev.med.60.041807.132435

[5] Wang AZ, Langer R, Farokhzad OC (2012) Nanoparticle delivery of cancer drugs. Annu Rev Med 63: 185–198. 21888516 10.1146/annurev-med-040210-162544

[6] Sawant RR, Torchilin VP (2012) Challenges in development of targeted liposomal therapeutics. AAPS J 14: 303–315. 22415612 10.1208/s12248-012-9330-0 PMC3326155

[7] Davis ME, Chen Z, Shin DM (2008) Nanoparticle therapeutics: an emerging treatment modality for cancer. Nat Rev Drug Discov 7: 771–782. 18758474 10.1038/nrd2614

[8] Park K (2007) Nanotechnology: What it can do for drug delivery. J Control Release 120: 1–3. 17532520 10.1016/j.jconrel.2007.05.003 PMC1949907

[9] Torchilin VP (2006) Recent Approaches to Intracellular Delivery of Drugs and DNA and Organelle Targeting. Annu Rev Biomed Eng 8: 343–375. 16834560 10.1146/annurev.bioeng.8.061505.095735

[10] Shao K, Hou Q, Duan W, Go ML, Wong KP, et al (2006) Intracellular drug delivery by sulfatide-mediated liposomes to gliomas. J Control Release 115: 150–157. 16963144 10.1016/j.jconrel.2006.07.024

[11] Shao K, Hou Q, Go ML, Duan W, Cheung NS, et al (2007) Sulfatide-tenascin interaction mediates binding to the extracellular matrix and endocytic uptake of liposomes in glioma cells. Cell Mol Life Sci 64: 506–515. 17279316 10.1007/s00018-007-6419-1 PMC11138434

[12] Townson K, Greenshields KN, Veitch J, Nicholl D, Eckhardt M, et al (2007) Sulfatide binding properties of murine and human antiganglioside antibodies. Glycobiology 17: 1156–1166. 17855742 10.1093/glycob/cwm095

[13] He H, Nilsson CL, Emmett MR, Ji Y, Marshall AG, et al (2010) Polar lipid remodeling and increased sulfatide expression are associated with the glioma therapeutic candidates, wild type p53 elevation and the topoisomerase-1 inhibitor, irinotecan. Glycoconj J 27: 27–38. 19557511 10.1007/s10719-009-9249-6

[14] Brellier F, Chiquet-Ehrismann R (2012) How do tenascins influence the birth and life of a malignant cell? Journal of Cellular and Molecular Medicine 16: 32–40. 21692981 10.1111/j.1582-4934.2011.01360.x PMC3823091

[15] De Santis R, Albertoni C, Petronzelli F, Campo S, D'Alessio V, et al (2006) Low and high tenascin-expressing tumors are efficiently targeted by ST2146 monoclonal antibody. Clin Cancer Res 12: 2191–2196. 16609034 10.1158/1078-0432.CCR-05-2526

[16] Mukaratirwa S, Koninkx JF, Gruys E, Nederbragt H (2005) Mutual paracrine effects of colorectal tumour cells and stromal cells: modulation of tumour and stromal cell differentiation and extracellular matrix component production in culture. Int J Exp Pathol 86: 219–229. 16045544 10.1111/j.0959-9673.2005.00425.x PMC2517436

[17] Stewart JCM (1980) Colorimetric determination of phospholipids with ammonium ferrothiocyanate. Anal Biochem 104: 10–14. 6892980 10.1016/0003-2697(80)90269-9

[18] Alvarez-Cedron L, Sayalero ML, Lanao JM (1999) High-performance liquid chromatographic validated assay of doxorubicin in rat plasma and tissues. J Chromatogr B Biomed Sci Appl 721: 271–278. 10052699 10.1016/s0378-4347(98)00475-7

[19] Urva SR, Shin BS, Yang VC, Balthasar JP (2009) Sensitive high performance liquid chromatographic assay for assessment of doxorubicin pharmacokinetics in mouse plasma and tissues. Journal of Chromatography B-Analytical Technologies in the Biomedical and Life Sciences 877: 837–841. 10.1016/j.jchromb.2009.02.018 19246257

[20] Zhang FY, Du GJ, Zhang L, Zhang CL, Lu WL, et al (2009) Naringenin enhances the anti-tumor effect of doxorubicin through selectively inhibiting the activity of multidrug resistance-associated proteins but not P-glycoprotein. Pharm Res 26: 914–925. 19067124 10.1007/s11095-008-9793-y

[21] Jung SH, Jung SH, Seong H, Cho SH, Jeong KS, et al (2009) Polyethylene glycol-complexed cationic liposome for enhanced cellular uptake and anticancer activity. Int J Pharm 382: 254–261. 19666094 10.1016/j.ijpharm.2009.08.002

[22] Elbayoumi TA, Torchilin VP (2009) Tumor-targeted nanomedicines: enhanced antitumor efficacy in vivo of doxorubicin-loaded, long-circulating liposomes modified with cancer-specific monoclonal antibody. Clin Cancer Res 15: 1973–1980. 19276264 10.1158/1078-0432.CCR-08-2392 PMC2762655

[23] Xiong XB, Huang Y, Lu WL, Zhang X, Zhang H, et al (2005) Enhanced intracellular delivery and improved antitumor efficacy of doxorubicin by sterically stabilized liposomes modified with a synthetic RGD mimetic. J Control Release 107: 262–275. 16125816 10.1016/j.jconrel.2005.03.030

[24] Li X, Ding L, Xu Y, Wang Y, Ping Q (2009) Targeted delivery of doxorubicin using stealth liposomes modified with transferrin. Int J Pharm 373: 116–123. 19429296 10.1016/j.ijpharm.2009.01.023

[25] Adams E (1948) A method for counting blood platelets in small animals. Yale J Biol Med 21: 17–20. 18122096 PMC2598795

[26] Oliveira RAG, Takadachi MM, Nonoyama K, Barretto OCdO (2003) The absolute recommendation of chamber Neubauer method for platelets counting instead of indirect methods in severe thrombocytopenic patients. Rio de Janeiro 39.

[27] Singal PK, Iliskovic N (1998) Doxorubicin-induced cardiomyopathy. N Engl J Med 339: 900–905. 9744975 10.1056/NEJM199809243391307

[28] Herman EH, Zhang J, Lipshultz SE, Rifai N, Chadwick D, et al (1999) Correlation between serum levels of cardiac troponin-T and the severity of the chronic cardiomyopathy induced by doxorubicin. J Clin Oncol 17: 2237–2243. 10561281 10.1200/JCO.1999.17.7.2237

[29] Koh E, Nakamura T, Takahashi H (2004) Troponin-T and brain natriuretic peptide as predictors for adriamycin-induced cardiomyopathy in rats. Circ J 68: 163–167. 14745153 10.1253/circj.68.163

[30] Wang J, Goh B, Lu W, Zhang Q, Chang A, et al (2005) In vitro cytotoxicity of Stealth liposomes co-encapsulating doxorubicin and verapamil on doxorubicin-resistant tumor cells. Biol Pharm Bull 28: 822–828. 15863886 10.1248/bpb.28.822

[31] Li B, Xu H, Li Z, Yao M, Xie M, et al (2012) Bypassing multidrug resistance in human breast cancer cells with lipid/polymer particle assemblies. Int J Nanomedicine 7: 187–197. 22275834 10.2147/IJN.S27864 PMC3263411

[32] Allen TM, Cheng WWK, Hare JI, Laginha KM (2006) Pharmacokinetics and Pharmacodynamics of Lipidic Nano-Particles in Cancer. Anticancer Agents Med Chem 6: 513–523. 17100556 10.2174/187152006778699121

[33] Cabanes A, Even-Chen S, Zimberoff J, Barenholz Y, Kedar E, et al (1999) Enhancement of antitumor activity of polyethylene glycol-coated liposomal doxorubicin with soluble and liposomal interleukin 2. Clin Cancer Res 5: 687–693. 10100723

[34] Unezaki S, Maruyama K, Takahashi N, Koyama M, Yuda T, et al (1994) Enhanced delivery and antitumor activity of doxorubicin using long-circulating thermosensitive liposomes containing amphipathic polyethylene glycol in combination with local hyperthermia. Pharm Res 11: 1180–1185. 7971721 10.1023/a:1018949218380

[35] Accardo A, Salsano G, Morisco A, Aurilio M, Parisi A, et al (2012) Peptide-modified liposomes for selective targeting of bombesin receptors overexpressed by cancer cells: a potential theranostic agent. Int J Nanomedicine 7: 2007–2017. 22619538 10.2147/IJN.S29242 PMC3356180

[36] Xu L, Anchordoquy T (2011) Drug delivery trends in clinical trials and translational medicine: challenges and opportunities in the delivery of nucleic acid-based therapeutics. J Pharm Sci 100: 38–52. 20575003 10.1002/jps.22243 PMC3303188

[37] Wu J, Lu Y, Lee A, Pan X, Yang X, et al (2007) Reversal of multidrug resistance by transferrin-conjugated liposomes co-encapsulating doxorubicin and verapamil. J Pharm Pharm Sci 10: 350–357. 17727798

[38] Oudard S, Thierry A, Jorgensen TJ, Rahman A (1991) Sensitization of multidrug-resistant colon cancer cells to doxorubicin encapsulated in liposomes. Cancer Chemother Pharmacol 28: 259–265. 1678995 10.1007/BF00685532

[39] Manil L, Couvreur P, Mahieu P (1995) Acute renal toxicity of doxorubicin (adriamycin)-loaded cyanoacrylate nanoparticles. Pharm Res 12: 85–87. 7724492 10.1023/a:1016290704772

[40] Charrois GJ, Allen TM (2003) Rate of biodistribution of STEALTH liposomes to tumor and skin: influence of liposome diameter and implications for toxicity and therapeutic activity. Biochim Biophys Acta 1609: 102–108. 12507764 10.1016/s0005-2736(02)00661-2

[41] Campbell RB (2006) Tumor Physiology and Delivery of Nanopharmaceuticals. Anticancer Agents Med Chem 6: 503–512. 17100555 10.2174/187152006778699077

[42] Drummond DC, Meyer O, Hong K, Kirpotin DB, Papahadjopoulos D (1999) Optimizing liposomes for delivery of chemotherapeutic agents to solid tumors. Pharmacol Rev 51: 691–743. 10581328

[43] Alexis F, Pridgen E, Molnar LK, Farokhzad OC (2008) Factors Affecting the Clearance and Biodistribution of Polymeric Nanoparticles. Mol Pharm 5: 505–515. 18672949 10.1021/mp800051m PMC2663893

[44] Oskarsson T, Acharyya S, Zhang XH, Vanharanta S, Tavazoie SF, et al (2011) Breast cancer cells produce tenascin C as a metastatic niche component to colonize the lungs. Nat Med 17: 867–874. 21706029 10.1038/nm.2379 PMC4020577

[45] Miura R, Aspberg A, Ethell IM, Hagihara K, Schnaar RL, et al (1999) The proteoglycan lectin domain binds sulfated cell surface glycolipids and promotes cell adhesion. J Biol Chem 274: 11431–11438. 10196237 10.1074/jbc.274.16.11431

[46] Pesheva P, Gloor S, Schachner M, Probstmeier R (1997) Tenascin-R is an intrinsic autocrine factor for oligodendrocyte differentiation and promotes cell adhesion by a sulfatide-mediated mechanism. J Neurosci 17: 4642–4651. 9169525 10.1523/JNEUROSCI.17-12-04642.1997 PMC6573339

