# Supplementary figures and images for: Enhanced Antitumor Efficacy and Reduced Systemic Toxicity of Sulfatide-Containing Nanoliposomal Doxorubicin in a Xenograft Model of Colorectal Cancer
Source: PLoS One. 2012 Nov 7;7(11):e49277. doi: 10.1371/journal.pone.0049277 (PMC3492268; doi:10.1371/journal.pone.0049277)

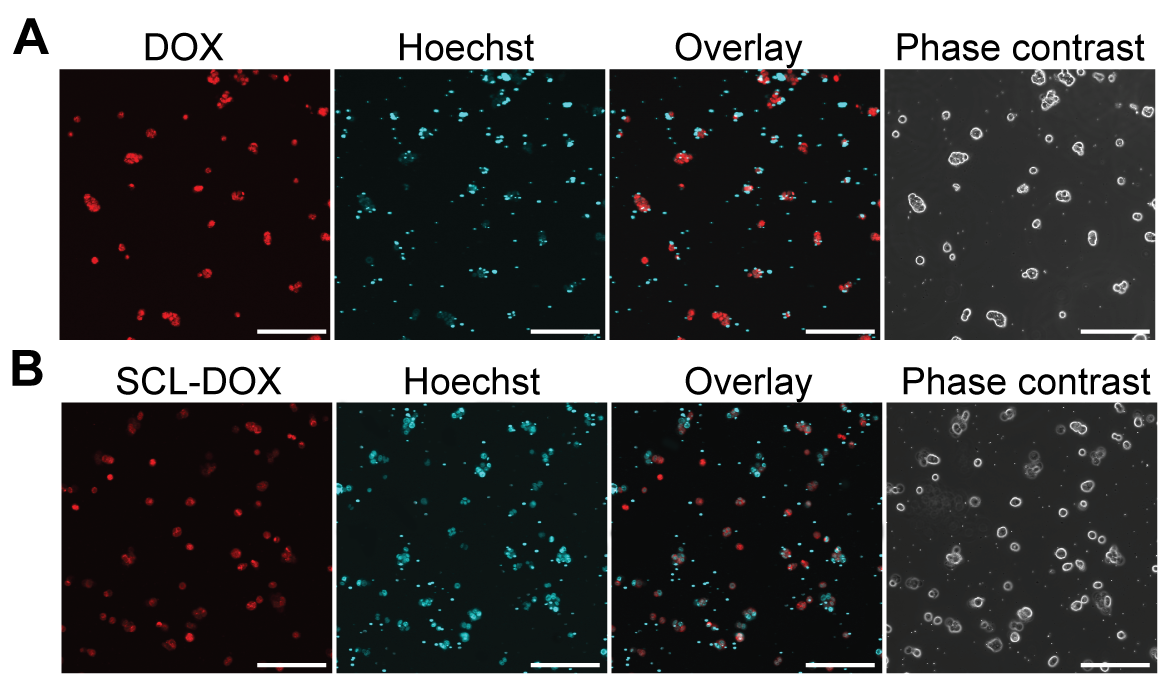

Supplement: Figure S1 — Intracellular uptake of SCL-DOX in HT-29 cells. HT-29 cells were incubated with 2 µg/mL free DOX or equivalent SCL-DOX for 24 h. Following two washes with PBS, cells were imaged with a confocal fluorescence microscope. (A) Cells treated with free DOX. (B) Cells treated with SLC-DOX. Red: fluorescence from DOX; blue: nuclei stained with Hoechst 33342. Scale bars: 200 µm. (TIF) [file pone.0049277.s001.tif]

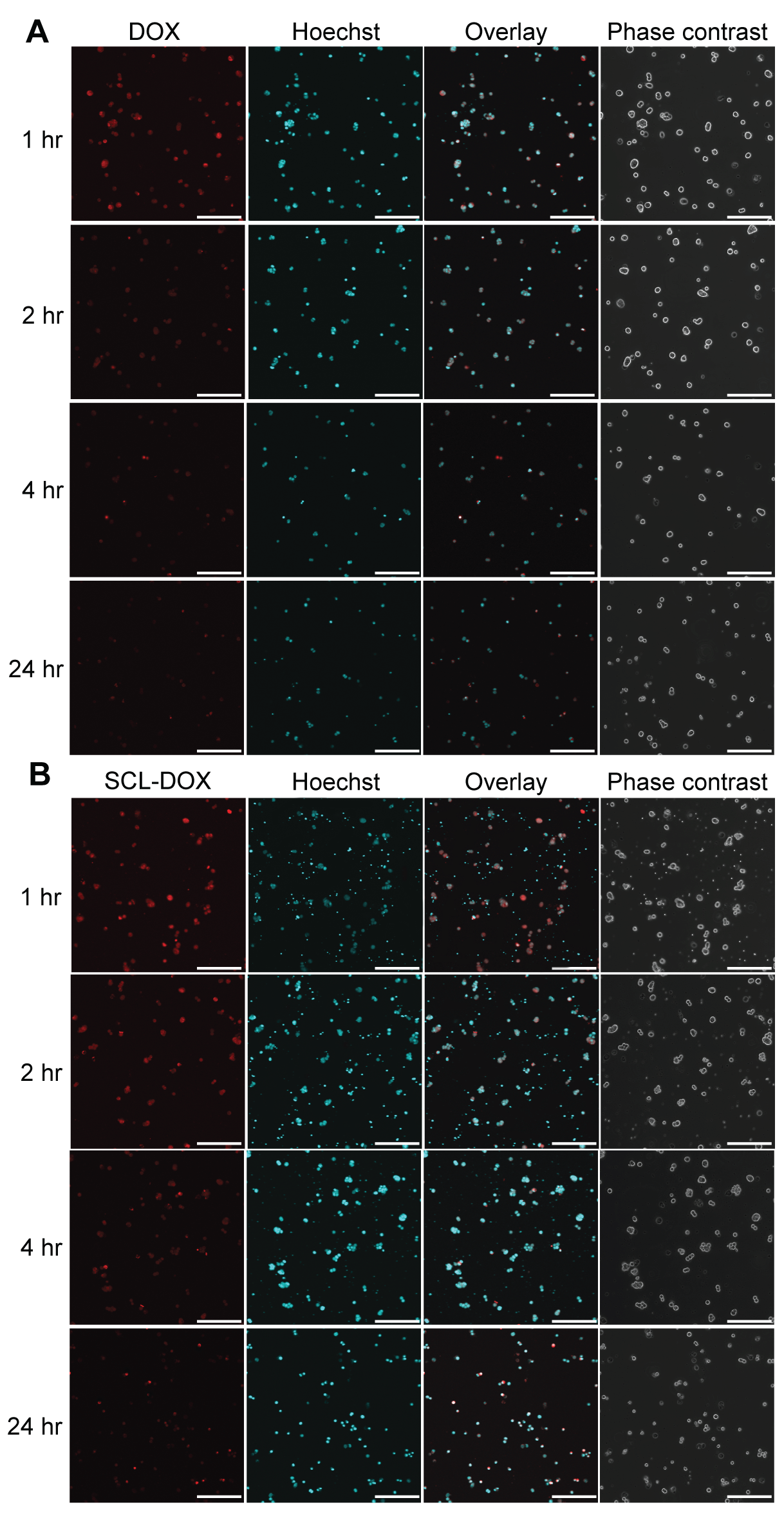

Supplement: Figure S2 — Intracellular retention of SCL-DOX in HT-29 cells. HT-29 cells were first incubated with 2 µg/mL free DOX or equivalent SCL-DOX for 24 h. After two washes with PBS to remove the drugs, cells were cultured in fresh full culture medium followed by imaging serially at 1 h, 2 h, 4 h and 24 h using fluorescence confocal microscopy. (A) Cells treated with free DOX. (B) Cells treated with SLC-DOX. Red: fluorescence from DOX; blue: nuclei stained with Hoechst 33342. Scale bars: 200 µm. (TIF) [file pone.0049277.s002.tif]
